# Correlation between HBsAg quantitative assay results and HBV DNA levels in chronic HBV

**Published:** 2011-05-01

**Authors:** Azita Ganji, Abbas Esmaeilzadeh, Kamran Ghafarzadegan, Hoda Helalat, Houshang Rafatpanah, Ali Mokhtarifar

**Affiliations:** 1 Department of Internal Medicine , Imam Reza Hospital, Mashhad University of Medical Sciences, Mashhad, IR Iran; 2 Department of Pathology, Cancer Research Center, Mashhad, IR Iran; 3 Immunology Research Center, School of Medicine, Mashhad University of Medical Sciences, Mashhad, IR Iran

**Keywords:** Chronic hepatitis B, Quantitative HBsAg, HBV DNA level

## Abstract

**Background:**

Viral load has been used to diagnose and monitor patients who are being treated for chronic hepatitis B (CHB). The Diagnosis methods are molecular-based and expensive. Quantitation of hepatitis B surface antigen (HBsAg) by automated chemiluminescent micro-particle immunoassay has been proposed to be a surrogate marker. Quantitating HBV DNA levels molecularly is expensive; thus, a cheaper laboratory test as a surrogate diagnostic marker might simplify our management.

**Objectives:**

We determined whether quantitative HBsAg levels correlate with HBV DNA levels in CHB.

**Patients and Methods:**

In this cross-sectional study, all CHB patients who were referred by a gastroenterologist to undergo quantitative HBV DNA assay in a qualified laboratory in Mashhad, Iran in 2009 were enrolled, and blood samples was obtained. Patients who were positive for antibodies to HCV and HDV were excluded. HBV DNA was measured by real-time polymerase chain reaction, and serum HBsAg was quantified byelectrochemiluminescence assay (Roche Diagnostic).

**Results:**

Of 97 patients, 70 were male (72%) and 27 were female (28%); the mean age was 39 ± 11 years. Eighty-seven percent wasHBeAg-negative. By Mann-Whitney test,HBSAg titer differed significantly between HBeAg-positive and -negative patients (P = 0.001), as did HBV DNA levels (P = 0.009). By Spearman test, there was no significant correlation between HBsAg and HBV DNA levels (P= 0.606 and r = 0.53).

**Conclusions:**

HBeAg-negative patients have higher levels of HBsAg and lower levels of HBV DNA. By electrochemiluminescence assay,HBsAg has no significant correlation with HBV DNA levels in CHB with predominant genotype D and HBeAg negativity in Iran.

## Introduction

### Background

Chronic hepatitis B is a major global problem, affecting more than 350 million chronic Hepatitis B worldwide [1] and leading to 1 million deaths each year [2] Quantitative levels of HBV DNA, ALT levels, and histological findings are three factors to consider when determining HBV treatment. Active viral infection scan be detected by quantifying HBV DNA, but such assays are molecular-based and expensive. Considering the distribution of HBV, particularly in developing countries, a cheaper laboratory test that can be used as a surrogate marker for the molecular detection of HBV DNA might make our management more practical. Hepatitis B virus is a DNA virus that has a circular and partially double-stranded genome, which encodes four major proteins-S, P, C, X. HBsAg is the chief protein of the viral envelop, and serological assays that detect HBsAg have guided the diagnosis of hepatitis B infection. We hypothesize that it can be a useful tool for managing patients as well.

Recently, the relationship between serum HBsAg concentrations and HBV DNA levels in hepatitis B patients who are positive for serum HBsAg and HBeAg was examined. Serum HBsAg concentration was related to HBV DNA replication level; nevertheless, it is not feasible to use HBsAg concentration to monitor HBV replication levels [3]. In noncirrhotic patients, HBV DNA and HBsAg levels correlate negatively. HBsAg levels are low in HBeAg-positive patients but higher in HBeAg-negative cases; HBV DNA levels are higher in HBeAg-positive patients compared with HBeAg-negative cases [4]. In another study, however, serum HBsAg levels, using Architect HBsAg QT, were higher in HBeAg-positive than in anti-HBe positive chronic HBV carriers, correlating with the level of serum HBV DNA [5]. Quantitative measurements of HBsAg titer constitute a simple and economical reference for HBV replication in HBV carriers as well [6]. Previous studies have suggested that quantitative hepatitis B surface antigen (HBsAg) is also a surrogate marker that can be used to monitor patients with CHB who are being treated, and HBsAg titer is related to HBV DNA levels [7].

### Objectives

Using serum HBsAg concentration as a marker of HBV replication level in hepatitis B patients, we determined whether quantitative HBsAg correlates with hepatitis B virus (HBV) DNA levels in CHB in Iran.

## Materials and Methods

This descriptive analytical study (cross-sectional) was performed to determine the correlation of serum HBSAg level and quantitative HBV DNA level in patients with chronic hepatitis B in the Department of Gastroenterology and Hepatology, Imam Reza Hospital, Mashhad, Iran. All CHB patients who were HBsAg-positive for more than 6 months and referred by a hepatologist to a qualified virology laboratory in Mashhad to undergo HBV DNA assay, were enrolled and selected by nonrandom sampling.

After the purpose of research was explained and informed consent was obtained, samples were drawn and HBsAg, HCV Ab, HDV Ab, HBeAg, AST, and ALT levels were measured. Those who were positive for antibodies to HCV and HDV were excluded.HBV DNA was measured by real-time PCR, and based on the detection limits, viral loads that were less than 100 was considered undetectable and excluded. ALT levels were measured by ELISA. Serum HBsAg was quantified by electrochemiluminescence assay (Roche Diagnostic), wherein HBsAg was expressed in IU/ml. One hundred fifteen patients with chronic hepatitis B with HBeAg positive or negative were enrolled, 14 of whom were HDV-positive; and totally we excluded 4 patients due to missing data. SPSS 16 was used to analyze the data. Spearman correlation coefficient was used to correlate serum levels of HBsAg and HBV DNA levels. P ≤ 0.05 was considered significant.

## Results

### Statistical Analysis

**Figure 1:**
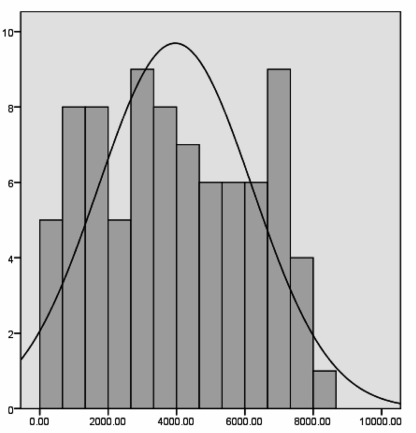
HBsAg titer in CHB (Mean = 3962.63, Std. Dev. = 2250.078, N = 82)

**Figure 2:**
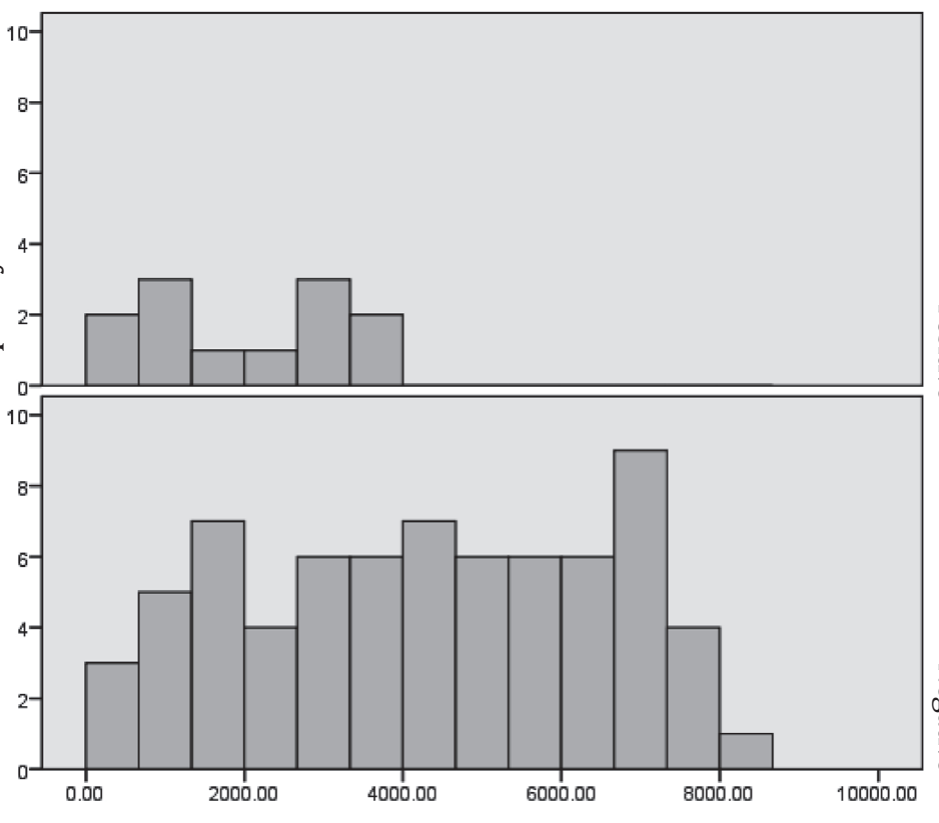
Frequency of HBsAg titer in HBeAg-positive and -negative CHB

Fourteen patients (12%) with CHB were HDV-positive and excluded. Ninety-seven of 115 patients with CHB were included; 70 were male (72%) and 27 were female(28%). The mean age was 39 ± 11 years. Eighty-five CHB patients were HBeAg-negative (87%) and 12 (13%) were HBeAg-positive. By Kolmogorov-Smimovtest, the distribution of HBsAg titers was normal and irregular for HBV DNA levels, HBeAg titer, AST, and ALT. Mean HBsAg level was 4021 ± 2305 IU/ml;mean AST level was 54 ± 94 U/L and mean ALT was 62 ± 84 U/L. Mean HBV DNA level was 4.69×106 ± 1.839× 10(7) copy/ml (Figure 1).By Mann-Whitney test, HBSAg titer differed significantly between HBeAg-positive and -negative patients (P = 0.001). In 12 HBeAg-positive patients, less than 25% had HBsAg titer = 1088 (Q1 = 1088) and HBsAg titer in 25-50% of patients (Q2) = 2030 and in more than 75% of patients (Q3), HBsAg titer were =3143 IU/ml and in 72 patients with HBeAg negative, HBsAg titer were in Q1= 2349, Q2 = 4369, and Q3 = 6108 IU/ml (Figure 2).

HBV DNA level also differed significantly between HBeAg-positive and negative patients (P = 0.009). HBV DNA level in HBeAg-positive subjects was Q1: 8.65×10³, Q2: 9.02 ×105 and Q3: 8.38×107 and in HBeAg negatives were: Q1 (less than 25% of patients) = 515, Q2 (25-50% of patients) = 3350 AND Q3 (more than 75% of patients) = 7/60 ×10 4.By Spearman test, there was no significant correlation between HBsAg and HBV DNA (P = 0.606 and r = 0.53). After the data were split, this correlation remained insignificant inHBeAg-positive (P = 0.053 and r = -0.57) and-negative patients (P = 0.605 and r = 0.057). There was no significant link between HBsAg level and ALT level (P = 0.45). HBV DNA levels correlated with ALT (P = 0.05, r = 0.19).

## Discussion

We attempted to correlate quantitative HBsAg levels with HBV DNA level in an area in Iran that is predominantly HBeAg-negative, in which nearly all patients are infected by genotype D [8]. HBsAg is a classical marker of infection with hepatitis B virus, and serological assays to detect HBsAg have guided its diagnosis. We hypothesized that HBsAg can be used to manage and monitor patients as well. On infection with HBV, closed circular DNA of HBV genome forms inside the nuclei of hepatocytes [9]. DNA-containing nucleocapsids become enveloped and are secreted into the blood [10]. Hepatitis B virus ccc DNA is a template for viral replication, correlating robustly with levels of total intracellular HBV DNA, serum HBV DNA, and HBsAg [11]. HBsAg quantification indirectly reflects the number of infected hepatocytes [12].

Chan, et al. observed that low pretreatment HBsAg is better than HBV DNA in predicting good responses to treatment [11]. In another study of HBeAg-negative CHB, however, HBsAg correlated poorly with serum HBV DNA and did not correlate with intrahepatic ccc DNA or total HBV DNA. Quantitative Immunohistochemistry for hepatocyte HBsAg confirmed its relationship with viral replication only in HBeAg-positive patients [13]. In our cases, 87% of whom were HBeAg-negative, there was no significant correlation between HBsAg level and HBV DNA level-even after subdivision into HBeAg-positive and -negative patients. Nevertheless, our analysis provides insight into the differences inHBsAg levels between HBeAg-positive and -negative patients, which appear to be affected by HBeAg status. HBV DNA levels were higher in HBeAg-positive patients, but HBsAg levels were higher in HBeAg-negative patients. In a previous study, we observed patent differences in HBs levels based on HBeAg status [14].

Studies have measured HBsAg concentration during the treatment of HBeAg-negative patients. The lowest rate of Sustained Virological Response (SVR) with PEG interferon was observed in those with genotype D [15], which experienced the smallest decline in HBsAg concentration during treatment [16] and had the lowest chance of a sustained response with interferon-alpha [17]. Thus, there are differences in the importance of quantitative HBsAg concentration for genotype D at baseline and during treatment. All HBV isolates in Iranian patients are genotype D [8]. We have an unusual subtype, "ayr," of the virus in Iran, which is not typical for HBV genotype D [18]. Based on previous studies in throughout Iran, all CHB patients in Iran have genotype D; thus, there was no need to determine the genotype.

In our study, we found statistically significant differences in HBsAg and HBV DNA levels between HBeAg-positive and -negative patients, but our sample size was likely too small to observe any significant correlation between HBsAg and HBV DNA levels. Conversely, it can be due to genotype D in Iran. In HBeAg-positive patients, HBsAg correlated with serum HBV DNA, intrahepatic ccc DNA, and total HBV DNA, but these correlations were poor in HBeAg-negative cases [13]. It appears thata cutoff value of 1500 IU/ml for serum HBsAg during treatment can be a predictor of seroconversion [19]. In other studies, serum HBsAg concentration has been used as a surrogate marker of HBV DNA in HBeAg-positive patients and those with high ALT; in their study, HBsAg titer predicted HBV DNA levels [20]. In HBeAg-negative patients, HBsAg levels were higher in patients with active disease than in those with inactive disease, and HBsAg remained stable in HBeAg-positive patients but tended to fall gradually in HBeAg-negative patients [21]. In our study, after dividing patients by active and inactive CHB (based on ALT > 50 and HBV DNA > 2000), we observed was no significant difference between active and inactive CHB, likely due to the small sample size.

HBsAg titer can help differentiate active from inactive carriers. Single-point combined HBsAg and HBV DNA quantification provide the most accurate identification of inactive carriers [22]. Thus, we need more studies with larger samples in active and inactive CHB to determine whether there is any correlation in this case.

There are 2 forms of HBsAg-one over intact virion, which includes small, medium, and large proteins in envelops and is related to viral infectivity, and another that exists as subviral particles in serum and is produced in great excess. These are predominantly S protein and, to a lesser extent, M and L protein; these are not infectious but are strongly immunogenic, stimulating antibody production. We propose that if we can detect 2 forms of HBsAg (over intact virion and subviral particle separately and quantitate them, we can determine the relationship between special forms of HBsAg and HBV DNA level.Based on our results, quantitative HBsAg level cannot be used as a surrogate marker for replicative state inHBeAg-negative patients in Iran. We recommend performing larger studies in Iran in different groups of CHB and patients who are being treated for CHB separately. HBeAg-negative patients have higher levels of HBsAg and lower levels of HBV DNA. by electrochemiluminescence assay do not correlate significantly with HBV DNA level in HBeAg-positive or negative patients in Iran, necessitating additional studies to standardize quantification assays and define thresholds of HBsAg that have clinical predictive value.
